# The effect of vitamin D deficiency on the retinal microvasculature: an observational case-control study

**DOI:** 10.5935/0004-2749.20220089

**Published:** 2025-02-11

**Authors:** Müjdat Karabulut, Sinem Karabulut, Tugba Dübektaş Canbek, Sabahattin Sül, Aylin Karalezli

**Affiliations:** 1 Mugla Sıtkı Koçman University Medical School, Department of Ophthalmology, Mugla, Turkey; 2 Mugla Sıtkı Koçman University Medical School, Department of Internal Medicine, Mugla, Turkey

**Keywords:** Vitamin D deficiency, Retinal vessels/physiopathology, Vascular diseases/prevention & control, Tomography, optical coherence, Deficiência de vitamina D, Vasos retinianos/fisiopatologia, Doenças vasculares/ prevenção & controle, Tomografia de coerência óptica

## Abstract

**Purpose:**

To determine the effects of vitamin D deficiency on retinal microvascularity using optical coherence tomography angiography. Methods: This study was designed as an observational case-control study. Ninety-eight eyes of patients with vitamin D deficiency and 96 eyes of healthy participants with serum vitamin D level >30 ng/mL were studied. Macula centered, 6.00 × 6.00 mm scan size images were taken. The vessel densities in the superficial and deep retinal capillary plexus, foveal avascular zone area, and choriocapillaris flow area were measured.

**Results:**

The groups were comparable in terms of best-corrected visual acuity, sex, axial length, refractive error, age, and adjusted intraocular pressure. The average vitamin D level was significantly lower in the study group (p=0.021). The whole, parafoveal, and perifoveal vessel densities in the deep capillary plexus were considerably higher in the study group than in the control group (p=0.012, p=0.014, and p=0.023, respectively). The foveal avascular zone area and the choriocapillaris flow area were similar in both groups (p=0.37 and p=0.27, respectively) there was a strong negative correlation between the serum vitamin D level and vessel density in the whole image, parafoveal, and perifoveal regions of the deep capillary plexus in the study group (Spearman’s rho=-0.71, p=0.043; Spearman’s rho= -0.79, p=0.011; and Spearman’s rho = -0.74, p=0.032; respectively).

**Conclusion:**

An increase in vessel density might originate from vascular structural changes caused by vitamin D deficiency. The increased vessel density, especially in the deep capillary plexus, can enable early diagnosis of vitamin D-associated vasculopathy.

## Abstract (pt)

**Objetivo:**

Determinar os efeitos da deficiência de vitamina D nos microvasos da retina usando angiotomografia de coerência óptica.

**Métodos:**

Este estudo foi planejado para ser do tipo caso-controle observacional. Foram avaliados 98 olhos de pacientes com deficiência de vitamina D e 96 olhos de participantes saudáveis com nível sérico de vitamina D superior a 30 ng/mL. Foram adquiridas imagens de varredura centralizadas na mácula, com um tamanho de 6,00 × 6,00 mm. Mediram-se a densidade dos vasos nos plexos capilares superficial e profundo da retina, a área da zona avascular foveal e a área do fluxo coriocapilar.

**Resultados:**

Os grupos mostraram-se semelhantes em relação à melhor acuidade visual corrigida, ao gênero, ao comprimento axial, ao erro refrativo, à idade e à pressão intraocular ajustada. O nível médio de vitamina D foi significativamente menor no grupo de estudo (p=0,021). As densidades total, parafoveal e perifoveal do plexo capilar profundo foram significativamente maiores no grupo de estudo que no grupo controle (respectivamente, p=0,012, p=0,014 e p=0,023). As áreas da zona avascular foveal e do fluxo coriocapilar foram semelhantes nos dois grupos (respectivamente, p=0,37 e p=0,27). Além disso, houve uma forte correlação negativa do nível sérico de vitamina D com as densidades vasculares medidas em toda a imagem e nas regiões parafoveais e perifoveais do plexo capilar profundo no grupo de estudo (respectivamente, ρ de Spearman = -0,71, p=0,043; ρ de Spearman = -0,79, p=0,011; e ρ de Spearman = -0,74, p=0,032).

**Conclusão:**

Pode ocorrer um aumento na densidade vascular da retina devido a alterações estruturais dos vasos causadas pela deficiência de vitamina D. O aumento da densidade vascular, especialmente no plexo capilar profundo, pode ser usado para o diagnóstico precoce da vasculopatia associada à deficiência de vitamina D.

## INTRODUCTION

Vitamin D deficiency is prevalent worldwide. About 50% of the global population exhibits lower vitamin D levels^(1,2)^. It is a fat-soluble prohormone that is initially produced in the skin because of contact with sunlight and converted into active vitamin D via various metabolic processes. The first hydroxylation by one or more cytochrome P450 happens in the liver, and 25-hydroxyvitamin D (25(OH)D), also known as calcidiol, is synthesized. After the second hydroxylation in the kidneys, calcidiol is transformed into 1,25-dihydroxy vitamin D3 (1,25(OH)2D), also called calcitriol, which is responsible for most biological actions^(3)^.

Because 25(OH)D is the major circulating vitamin D configuration, its serum level was recently considered the best vitamin D supply indicator in the body. The total 25(OH)D serum level ranges from 25 ng/mL to 80 ng/mL^(4)^.

Although the exact mechanisms via which vitamin D deficiency influences vascular diseases remain unknown, current evidence reveals a strong association between vitamin D deficiency and large-vessel diseases, such as atherosclerosis, arterial stiffness, and arterial stenosis^(5-7)^. Some studies have also demonstrated a relationship between vitamin D deficiency and some microvascular diseases, including poor coronary microcirculation, endothelial dysfunction, nephropathy, cerebral small-vessel disease, and retinal microvascular damage^(8-11)^.

Optical coherence tomography angiography (OCT-A) can be used to obtain images of the structural and functional details of the retinal microvasculature. We aimed to define the effect of vitamin D deficiency on retinal microvasculature using this novel technique.

## METHODS

**Figure 1:**
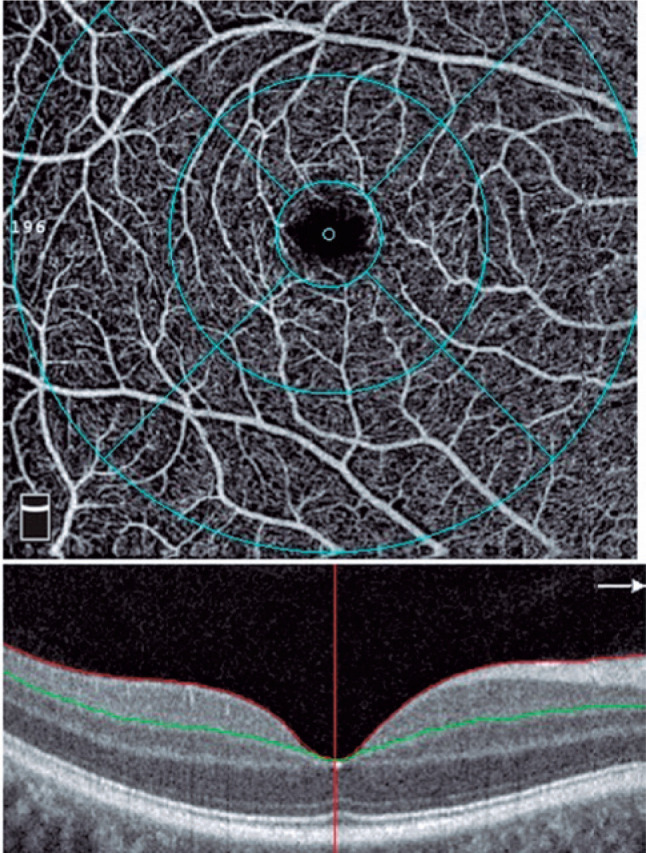
The SCP is located between the ILM (red line) and the IPL (green line) in a 6.00 × 6.00 mm macular scan size. Circles demonstrate the fovea, parafovea, perifovea, and whole image.

**Figure 2:**
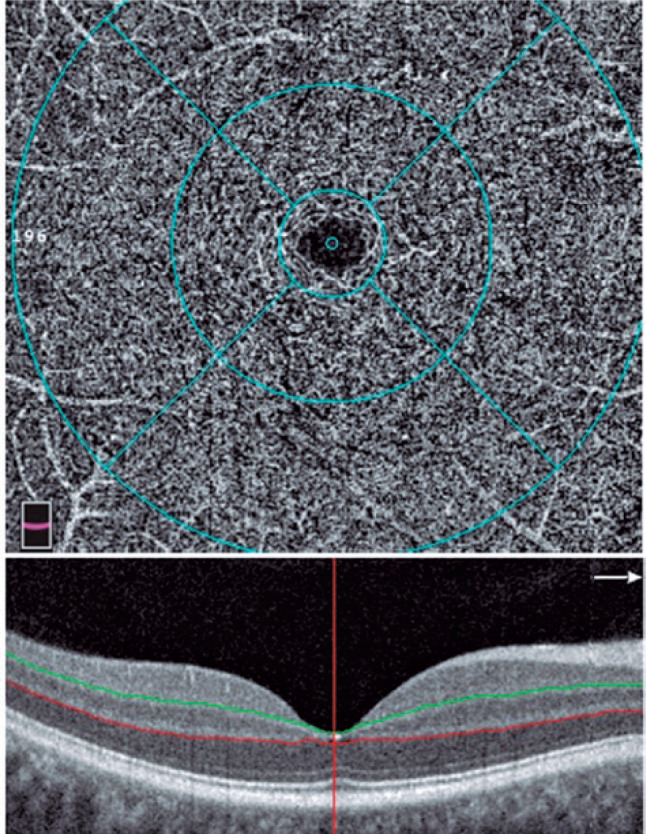
The DCP is located between the IPL (green line) and the OPL (red line) in a 6.00 × 6.00 mm macular scan size. Circles demonstrate the fovea, parafovea, perifovea, and whole image.

**Figure 3:**
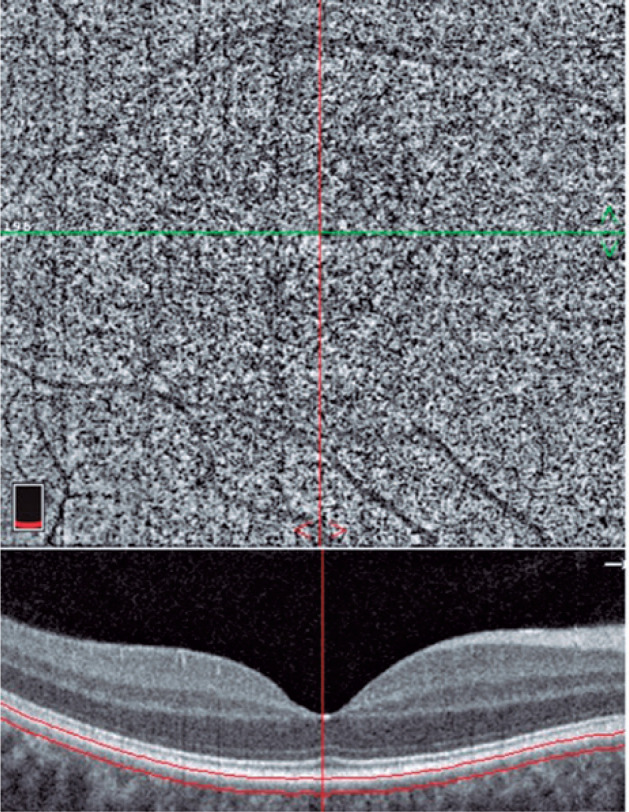
Choriocapillaris (between red lines) is located at a depth of 30 µm from the BRM.

**Figure 4:**
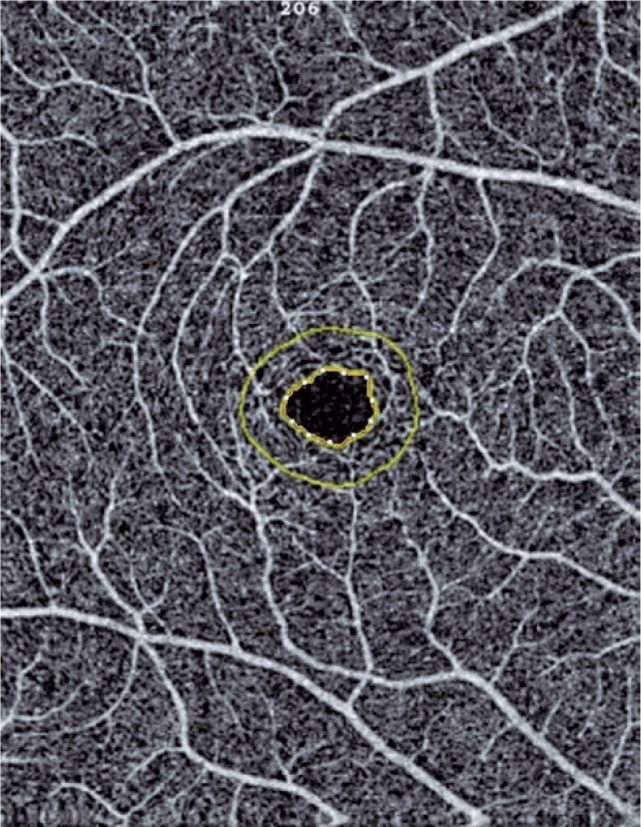
Foveal avascular zone in a 6.00 × 6.00 mm macular scan size.

Ninety-eight eyes of patients were compared to those of healthy controls. Patients who presented to the internal disease clinic for routine health control and whose vitamin S level was ≤20 ng/mL were enrolled in the study group. Individuals whose serum vitamin D levels were ≥30 ng/mL were allocated to the control group. Patients with retinal vascular diseases (i.e., any stage of diabetic and hypertensive retinopathy, senile maculopathy, and uveitis); any kind of nystagmus; a history of previous ocular surgery, amblyopia, glaucoma, or systemic diseases (i.e., diabetes, arterial hypertension, dyslipidemia, vasculitis, and rheumatologic and neurologic diseases); optic nerve disease; spherical power >3 diopters (D); cylindrical power >1.5 D; axial length (AL) >26 mm and <20 mm; and best-corrected visual acuity (BCVA) <1.00 decimal were excluded. In both groups, one eye that was eligible for inclusion was randomly selected. Patients whose body mass index was <18 kg/m^2^ and >25 kg/m^2^ and who were taking vitamin D analogs were also excluded. Images with artifacts and signal strength index <60 were not used. All patients provided informed consent. After a complete examination, macula-centered photos were automatically taken by a single expert who was blinded to the study using RTVue-XR Avanti (Optovue, Inc., CA, USA) on a 6.0 × 6.0-mm scan size. The vessel density (VD) in the superficial (SCP) and deep capillary plexus (DCP), choriocapillaris flow area, and foveal avascular zone (FAZ) area were measured (Figures 1, 2, 3, 4). OCT-A examinations and data were analyzed by an author who was blinded to the study groups. The OCT-A parameters were automatically calculated using the software embedded in the devices. The retinal microvasculature was analyzed using the automated retinal layer segmentation algorithm available on the device. In cases with insufficient automatic layer segmentation, the correction was manually performed.

SCP: Superficial capillary plexus, ILM: Internal limiting membrane, IPL: inner plexiform layer

DCP: Deep capillary plexus, IPL: inner plexiform layer, OPL: outer plexiform layer.

BRM: Bruch’s membrane.

Statistical Package for the Social Sciences (SPSS) 21.0.0.0 version (IBM Corporation and other(s) 1989, 2012) was used for data analyses. After proving the homogeneity and normal distribution of VD, FAZ area, choriocapillaris flow area, intraocular pressure (IOP), and AL value data with Shapiro-Wilk and Levene’s test (p>0.05 for VD, FAZ area, and choriocapillaris flow area, IOP, AL variables with all tests), an independent samples t-test was performed to compare the groups. Age, spherical power, and cylindrical power, and serum vitamin D levels were not distributed normally and homogeneously (p<0.05 for these variables); therefore, we used the Mann-Whitney U test to compare these parameters. Spearman’s correlation coefficient was used to determine the correlations between VD and serum vitamin D levels. Chi-square test was performed for sex-based comparisons. P<0.05 was considered to indicate statistical significance.

All the patients provided informed consent, and the study was conducted according to the principles in the Helsinki Declaration. The Local Clinical Research Ethics Committee approved this study (decision number: 02/ VI- date: 30/01/2020).

## RESULTS

The mean ± standard deviation serum vitamin D level was 15.3 ± 4.7 ng/mL and 34.6 ± 3.4 ng/mL in the study group and control group, respectively (p=0.021). The groups were similar regarding the average age, adjusted IOP for corneal thickness, AL, spherical and cylindrical power, and sex (p=0.10, p=0.12, p=0.65, p=0.29, p=0.15, x^^2^^(2, n = 194) = 5.8, and p=0.78) (Table 1). The mean BCVA in both the groups was 1.00-decimal. Although the VD in all regions of the SCP and the choriocapillaris flow area was increased in the study group (p=0.24, p=0.52, p=0.38, p=0.45, and p=0.27, respectively) (Table 2), there was a significant difference in the whole image, parafoveal, and perifoveal regions of the DCP in the study and control groups (p=0.012, p=0.014, and p=0.023, respectively) (Table 2). The FAZ area was smaller in the study group; however, the difference was insignificant (p=0.37) (Table 2). Moreover, there was a strong inverse relationship between the serum vitamin D levels and VD in the whole image, parafoveal, and perifoveal regions of the DCP (Spearman’s rho = -0.71, p=0.043; Spearman’s rho = -0.79, p=0.011; and Spearman’s rho = -0.74, p=0.032; respectively) (Table 3). The correlation between the serum vitamin D levels and VD in all regions of the SCP was negative; however, the correlation was not statistically significant (Spearman’s rho = -0.39, p=0.13; Spearman’s rho = -0.43, p=0.10; Spearman’s rho = -0.40, p=0.12; and Spearman’s rho = 0.20, p=0.94; respectively) (Table 3).

**Table 1:** Demographic characteristics and the mean best-corrected visual acuity of the study and control groups.

	Study (n=98) Control (n=96)		P-value
	Mean ± SD		
Levels of vitamin D (ng/mL)	15.3 ± 4.7	34.6 ± 3.4	**0.021**
Mean age (y)	44.3 ± 9.3	48.2 ± 5.3	0.10
IOP (mmHg)	16.2 ± 2.8	15.9 ± 3.1	0.12
Sex Male	50	47	0.78
Female	48	49	
Mean BCVA (Decimal)	1.00	1.00	
AL (mm)	23.78 ± 1.13	23.66 ± 1.01	0.65
Spherical Power (D)	1.23 ± 1.45	1.17 ± 1.39	0.29
Cylindrical Power (D)	0.65 ± 0.73	0.69 ± 0.71	0.15

BCVA= Best-corrected visual acuity, IOP= Intra ocular pressure; SD= Standard deviation; AL= Axial length; D= Diopter

**Table 2:** Comparison of vessel density, FAZ area, choriocapillaris flow area on a 6.00 × 6.00 mm macular scan size

	Study group (n=98)	Control group (n=96)	
**Density (%)**	**Mean**	**± SD**	**P-value**
Superficial			
Whole image	52.4 ± 3.3	51.3 ± 3.1	0.24
Parafovea	54.7 ± 3.1	53.4 ± 2.7	0.52
Perifovea	52.9 ± 3.3	51.8 ± 3.4	0.38
Fovea	20.1 ± 5.9	19.9 ± 4.8	0.45
Deep			
Whole image	60.4 ± 4.1	56.3 ± 5.2	**0.012**
Parafovea	62.1 ± 2.2	59.7 ± 2.1	**0.014**
Perifovea	62.06 ± 3.2	57.8 ± 4.2	**0.023**
Fovea	39.1 ± 5.3	38.3 ± 4.6	0.78
FAZ area (mm^2^)	0.296 ± 0.09	0.317 ± 0.14	0.37
Choriocapillaris flow area (mm^2^)	2.09 ± 0.08	2.04 ± 0.11	0.27

FAZ= Foveal avascular zone. SD: Standard deviation

**Table 3:** Correlations between the vessel density in the DCP and the SCP and the serum vitamin D level in the study group

		Density in the DCP			
		Whole image	Parafovea	Perifovea	Fovea
Serum vitamin D level	rho	**-0.71**	**-0.79**	**-0.74**	**-0.21**
	P	**0.043**	**0.011**	**0.032**	**0.93**
			**Density in the SCP**		
		**Whole image**	**Parafovea**	**Perifovea**	**Fovea**
Serum vitamin D level	rho	**-0.39**	**-0.43**	**-0.40**	**-0.20**
	P	**0.13**	**0.10**	**0.12**	**0.94**

DCP= Deep capillary plexus; SCP= Superficial capillary plexus; P= p-value; rho= Spearman’s rho.

## DISCUSSION

In this observational case-control study, we found an increase in the VD of the SCP, DCP, and choriocapillaris flow area in the study groupe; however, there was only an apparent difference in the whole image, parafoveal, and perifoveal regions of the DCP in the study group. Furthermore, there was a strong negative correlation between the serum vitamin D levels and VD in the whole image, parafoveal, and perifoveal regions of the DCP.

Vitamin D is crucial for bone and mineral homeostasis; however, its deficiency has been related to an increased prevalence of multiple diseases, such as osteoporosis, autoimmune diseases, cancers, and cardiovascular diseases^(12)^.

Vitamin D plays several biological functions in atherosclerosis, inflammation, angiogenesis, arterial stiffness, and calcification by affecting many cell types to maintain healthy vasculature^(13)^. The effective form of vitamin D acts as a nuclear hormone via the binding vitamin D receptor (VDR) that is produced in most cells, such as immune cells; osteoblasts; myocytes; vascular endothelial, myocardial, and vascular smooth muscle cells; pericytes; neurons; osteoblasts; adipose tissue; and retinal cells^(14)^.

It was reported that VDR was found in some retinal layers, such as ganglion cells, inner nuclear layer, retinal photoreceptor layer, and pigment epithelium layer^(15)^. VDR expression has also been reported in retinal vascular endothelial cells, vascular smooth muscle cells, and pericytes^(16)^.

Pericytes are perivascular-supporting cells that are located outside the vessels and play essential roles in angiogenesis, equilibrium of afresh forming blood vessels, and vascular development. Pericytes express a significantly higher level of VDR than vascular endothelial cells; therefore, vitamin D deficiency mostly affects the functions of the pericytes. In the retinal endothelial cell, vitamin D reduces vascular injury by inhibiting vascular smooth muscle proliferation and migration^(17)^. Additionally, vitamin D reduces the proliferation and migration of pericytes by inhibiting its pro-angiogenic properties^(16)^.

In animal models, the outer plexiform layer expressed prominent amounts of D-dependent Ca^+^2^^-binding protein and VDR than the inner nuclear layer, inner plexiform layer, and photoreceptor cells. Thus, it was suggested that vitamin D deficiency primarily affects this layer more than the other levels of the retina^(18)^.

Recent studies have revealed a relationship between vitamin D deficiency and some eye diseases, including dry eye syndrome, diabetic retinopathy (DR), glaucoma, myopia, and age-related macular degeneration (AMD).

Observational studies have reported controversial results regarding the relationship between vitamin D deficiency and AMD. In a meta-analysis of 11 observational studies, Annweiler et al.^(19)^ reported that vitamin D was significantly related to late AMD, but not early AMD. Conversely, Wu et al.^(20)^ concluded no evidence to indicate a relationship between vitamin D deficiency and AMD risk; moreover, some studies have revealed that higher dietary intake of vitamin D yielded smaller drusen sizes and reduced neovascular AMD progression^(21)^.

Vitamin D deficiency has also been linked with Zhang et al.^(22)^ reported an inverse correlation between vitamin D levels and DR in a meta-analysis of 14 observational studies. Aksoy et al.^(23)^ also proposed an inverse correlation between serum vitamin D levels and DR severity; however, other studies did not report such a correlation^(24,25)^. Additionally, some researchers have recommended the consideration of vitamin D deficiency as a potential risk factor for developing open-angle glaucoma, central retinal vein occlusion, and myopia progression^(26-28)^.

In their prospective population-based cohort study, Mutlu et al.^(11)^ observed that lowered serum vitamin D levels were related to retinal microvascular damage, such as narrowed arterioles and enlarged veins. They measured the retinal vascular calibers with fundus photographs centered on the optic disc.

To our knowledge, no study has investigated the early effects of vitamin D deficiency on retinal microvascularity using OCT-A. In this case-control study, we enrolled patients who did not have any other eye or systemic diseases. We observed significantly increased VD in the whole image, parafoveal, and perifoveal regions of the DCP in our study group. Additionally, we observed a negative correlation between the serum vitamin D levels and VD in the whole image and the parafoveal and perifoveal regions of the DCP.

This study was not designed to determine causality. Nevertheless, we thought that increased VD, especially in DCP, might result from early-stage, vitamin D deficiency-associated retinal microvascular damage and reversible proliferation of pericytes and vascular smooth muscle as well as endothelial cells. The increase in VD does not indicate neovascularization because we did not detect any sign of neovascularization on fundus examination and colored fundus photography of the participants. Moreover, we believed that DCP was most affected owing to the prominent expression of D-dependent Ca+2-binding protein and VDR in the retina’s outer plexiform layer, as mentioned above. The negative correlation could indicate that vitamin D deficiency-associated retinal vasculopathy worsens as the serum vitamin D level decreases.

This study is unique in that we analyzed the effect of pure vitamin D deficiency on retinal microvasculature and identified the retinal layer that is mostly affected using OCT-A. This novel, noninvasive imaging technique could be used for early detection of vitamin D insufficiency-dependent microvasculopathy and diseases, such as atherosclerosis and angiogenesis, arterial stiffness osteoporosis, autoimmune diseases, cancers, and especially, cardiovascular diseases.

This study has certain limitations. Although the groups were similar with respect to age, sex, BCVA, IOP, AL, and refractive error, the number of eyes studied was relatively small. The study group could be divided into subgroups, such as deficient, insufficient, sufficient, and high according to the serum vitamin D levels to distinguish variations in the subgroups; moreover, we did not consider the effect of vitamin D supplementation on these OCT-A findings.

Consequently, OCT-A could be used to detect the early stages of cardiovascular and cerebrovascular diseases related to vitamin D insufficiency using retinal imaging. The increased VD, especially in DCP, might be used as an indicator for determining this early microvascular damage. Further prospective cohort studies are necessary to determine the effects of vitamin D supplementation therapy on retinal microvascularity using OCT-A.
